# Supplementation of Pelleted Hazel (*Corylus avellana*) Leaves Decreases Methane and Urinary Nitrogen Emissions by Sheep at Unchanged Forage Intake

**DOI:** 10.1038/s41598-018-23572-3

**Published:** 2018-04-03

**Authors:** Shaopu Wang, Melissa Terranova, Michael Kreuzer, Svenja Marquardt, Lukas Eggerschwiler, Angela Schwarm

**Affiliations:** 1 ETH Zurich, Institute of Agricultural Sciences, Animal Nutrition, Universitaetstrasse 2, 8092 Zurich, Switzerland; 2 Agroscope, Tioleyre 4, 1725 Posieux, Switzerland

## Abstract

This study is the first to quantify the effects of hazel (*Corylus avellana*) leaves on methane and urinary nitrogen emissions, digestibility, nitrogen and the energy balance of ruminants. Four experimental pellets were produced with 0, 30% and 60% hazel leaves, the latter also with 4% polyethylene glycol. Hazel leaves gradually replaced lucerne. The diet was composed of the pellets and grass hay (80%: 20%). Six adult sheep were allocated to all four treatments in a 6 × 4 crossover design. Including hazel leaves did not affect the feed intake, but it decreased the apparent digestibility of organic matter and fibre, especially at the high level. Methane emission was reduced by up to 25 to 33% per day, per unit of intake and per unit of organic matter digested. Urinary nitrogen excretion decreased by 33 to 72% with increasing levels of hazel leaves. The treatment with polyethylene glycol demonstrated that tannins in hazel leaves caused significant shares of the effects. In conclusion, the current results indicated a significant potential of hazel leaves as forage for ruminants to mitigate methane and urinary nitrogen emissions. Even high dietary hazel leaf proportions were palatable. The lower digestibility needs to be compensated with easily digestible diet ingredients.

## Introduction

Methane (CH_4_) from the livestock sector was calculated to account for 28% of the total global anthropogenic emissions, and is predicted to rise further due to an increasing worldwide demand for meat, milk and other animal-source products^1,2^. Manipulating the rumen fermentation by nutritional approaches with the aim of reducing CH_4_ and nitrogen (N) emissions from ruminant husbandry at concomitantly unchanged or even improved feed intake and digestibility are among the most important goals of current animal nutrition research. Various nutritional attempts to mitigate CH_4_ emission from ruminants have been undertaken, as reviewed by Beauchemin *et al*.^1^ and Hristov *et al*.^3^. Special attention has been given to plants or extracts rich in plant secondary compounds (PSC), such as tannins, essential oils or saponins. A number of screenings provided promising plants of at least moderate nutritional value that are effective in mitigating noxious emissions^4–6^. Different from synthetic compounds, the public’s growing concern about food safety is met by PSC-based forages^7^.

Tannins are a complex group of polyphenolic PSC with widely varying molecular weights that are found in many representatives of the plant kingdom^8^. When provided at high dosages, tannins were predominantly found to be antinutritional because of their adverse effects on feed intake and nutrient utilisation^9^. In recent years, however, they have been recognised as useful phytochemicals when provided at moderate dosages. Tannins can be beneficial modulators of rumen microbial fermentation promoting metabolic protein supply, animal productivity and animal health^10,11^. The feeding of tannin-containing plants or extracts from such plants to ruminants was found at times, but not always, to substantially reduce enteric CH_4_ emissions^12–14^. The effectiveness varies depending on the source, structure and supplemented level of the tannins^5^. In addition, tannins bind to proteins and thus protect at least part of the dietary protein from being degraded in the rumen. These bonds are cleaved in the abomasum, and thus the protein becomes at least partially digestible in the small intestine or, otherwise, will be excreted with the faeces^14,15^. Feeding diets containing tannins, therefore, is an efficient means to reduce the amount of dietary N to be excreted via urine and with that the N emission potential of the manure^16,17^.

The shrub hazel (*Corylus avellana*) grows wild in Europe and western Asia, but it is also cultivated for its nuts. Its leaves are considered a potential means of antioxidant and antibacterial effects because of their richness in phenolic compounds^18,19^. Besides, among the plant materials tested in the extensive EU screening project “Rumen Up”^20^, hazel leaves were found *in vitro* to depress CH_4_ production (mmol/g dry matter (DM) incubated) by 25%. Another recent *in vitro* screening confirmed the CH_4_ mitigation potential of hazel leaves and indicated their concomitantly favourable forage potential^21^. Hazel leaves were also found to inhibit the proteolytic activity in rumen fluid^20^. However, the effect of replacing part of the diet by hazel leaves on intake, total tract digestibility and mitigation of CH_4_ and urinary N losses in live ruminant livestock has not yet been determined. In addition, it is unclear yet whether the active ingredients of the hazel leaves are indeed represented by the tannins or also by other PSC.

The objective of the present study was to determine the quantitative effects of replacing lucerne (*Medicago sativa*) with hazel leaves at different proportions in forage-only diets fed to adult sheep. The following predictions were made: (i) Hazel leaves contain PSC which affect digestion in a way that CH_4_ and urinary N formation are mitigated in a dose-dependent way. (ii) These changes happen without major adverse effects on intake, nutrient and energy utilisation. (iii) The main active ingredients in hazel leaves responsible for the observed effect are the tannins, rather than other PSC like flavonoids. For this purpose, polyethylene glycol (PEG), which binds to tannins and inactivates them^22,23^, was added to one of the diets.

## Results

### Chemical composition of the diet ingredients

The hazel leaves contained slightly more organic matter (OM) than lucerne (treatment 0) but clearly less crude protein (CP) (Table 1). The hay was even lower in CP content. Both pellet ingredients (hazel leaves and lucerne) were similar in contents of neutral detergent fibre (NDF) and acid detergent fibre (ADF), whereas the hay contained much higher levels of NDF and ADF. The lignification of the fibre was low in the hay, intermediate in the lucerne and high in the hazel leaves. The hazel leaves contained about 8% of total phenols (TP), whereof 76% were tannins, with almost equal proportions of condensed tannins (CT) and hydrolysable tannins (HT). Both lucerne and hay contained much less TP and were almost free of CT. In the experimental pellets, a variation in TP contents from 2 to 7% was achieved, and the range for the CT was from 0 to 2% and for the HT from 1 to 3%.

**Table 1:** Ingredients and chemical composition of the experimental diets*.

Hazel leaves (% of pellets)	Hay^1^	Hazel leaves	Experimental pellets^1^			
			0^2^	30	60	60 + PEG^3^
Pellet ingredients (% of dry matter (DM))						
Lucerne (% of total pellets)			100	70.8	41.5	37.9
Hazel leaves (% of total pellets)			0	29.2	58.5	58.3
Polyethylene glycol (PEG, % of total pellets)			0	0	0	3.8
Analysed composition (% of DM)						
Organic matter	92.7	92.9	85.6	88.1	90.0	90.8
Crude protein	7.0	12.4	20.2	17.9	15.7	14.8
Ether extract	1.4	1.1	1.9	1.9	1.6	1.5
Neutral detergent fibre	68.9	48.5	44.2	42.9	44.4	44.9
Acid detergent fibre	41.4	30.7	33.1	31.1	30.1	32.5
Acid detergent lignin	7.4	12.2	9.5	11.0	11.5	14.5
Gross energy (MJ/kg DM)	17.5	18.3	16.9	17.4	17.8	18.2
Total phenols	1.43	8.16	1.72	4.14	6.55	5.94
Non-tannin phenols	0.82	1.95	0.96	1.33	1.75	1.58
Total tannins	0.61	6.21	0.76	2.82	4.80	4.36
Condensed tannins	0.02	3.39	0.01	1.11	2.43	1.36
Hydrolysable tannins	0.59	2.82	0.74	1.71	2.37	3.00

^1^The ratio of hay to pellet was 20%: 80% in total dietary DM. ^2^Equivalent to the composition of lucerne. ^3^Analysis, especially of the phenols, might have been compromised by the presence of PEG. ^*^Each animal received additionally 30 g/day of a commercial mineral-vitamin mixture (Kroni 461 Ovipress, Kroni Locher, Altstätten, Switzerland) consisting, per kg, of Ca, 140 g; P, 70 g; Mg, 40 g; Na, 50 g; Zn, 7 g; Mn, 3.5 g; Fe, 2.65 g; I, 60 mg; Co, 30 mg; Se, 40 mg; vitamin A, 400,000 IU; vitamin D_3_, 80,000 IU; vitamin E, 2 g; vitamin B_1_, 0.4 g and biotine, 0.1 g.

### Intake and digestibility

The total DM intake of the sheep, expressed per day or per kg of metabolic body weight (BW^0.75^), did not differ among treatments. The same was true for the intake of the two diet components, pellets and hay. However, a small but significant increase in OM intake per unit of BW^0.75^ was observed with the diet containing 50% hazel leaves with or without PEG compared to the control with no hazel leaves (Table 2). Water consumption linearly (*P* < 0.05) declined with an increasing hazel leaf proportion. Following the differences in the composition of the pellets, the CP and ADF intake per unit of BW^0.75^ largely differed, becoming linearly (*P* < 0.001) smaller with increasing levels of hazel leaves. The apparent digestibility of OM, NDF and ADF was linearly (*P* < 0.001) declining; in the case of OM digestibility, the decline became larger (quadratic contrast at *P* < 0.05) with a higher hazel leaf proportion, where the means also differed (*P* < 0.001) from the other diets. The addition of PEG to the diets containing 50% hazel leaves improved (*P* < 0.001) apparent digestibility to values intermediate between the diets with 25% and 50% hazel leaves or even to the level of the 25% diet (NDF digestibility). No difference (*P* > 0.10) between treatments was observed in the body weight (BW) of the sheep and NDF intake per unit of BW^0.75^.

**Table 2:** Effect of hazel leaves on intake, nutrient digestibility and body weight of the sheep (n = 6).

Hazel leaves (% of diet)	Experimental diets				SEM	*P* values		
	0	25	50	50 + PEG		Diet	L^1^	Q^1^
Dry matter intake (g/day)								
Total	2182	2147	2174	2170	45.5	0.64	0.71	0.56
Pellets	1794	1762	1780	1792	36.4	0.73	0.52	0.59
Hay	388	385	394	378	14.5	0.50	0.48	0.88
Daily intake (g/kg body weight^0.75^)								
Drinking water	217^a^	187^ab^	157^b^	162^ab^	7.8	0.037	0.024	0.95
Dry matter	85.5	85.3	85.9	85.0	0.80	0.66	0.84	0.79
Organic matter	74.1^b^	75.9^ab^	77.7^a^	77.4^a^	0.77	0.006	<0.001	0.78
Crude protein	15.3^a^	13.6^b^	12.2^c^	11.4^d^	0.33	<0.001	<0.001	0.33
Neutral detergent fibre	41.5	40.5	41.8	41.6	0.47	0.19	0.30	0.022
Acid detergent fibre	29.5^a^	28.0^bc^	27.5^c^	28.9^ab^	0.34	<0.001	<0.001	0.091
Gross energy (MJ)	1.45^c^	1.48^bc^	1.52^ab^	1.54^a^	0.016	0.001	<0.001	0.98
Apparent digestibility (%)								
Organic matter	58.2^a^	55.5^a^	48.6^c^	51.5^b^	0.85	<0.001	<0.001	0.046
Neutral detergent fibre	49.0^a^	41.6^b^	31.2^c^	39.8^b^	1.46	<0.001	<0.001	0.32
Acid detergent fibre	45.2^a^	30.7^b^	11.8^d^	23.4^c^	2.60	<0.001	<0.001	0.15
Body weight (kg)	74.5	73.6	73.8	74.9	1.32	0.62	0.22	0.84

L, linear effect of hazel leaf proportion; PEG, polyethylene glycol; Q, quadratic effect of hazel leaf proportion; SEM, standard error of mean. Means carrying no common superscript are different at *P* < 0.05. ^1^For this analysis, only diets 0, 25 and 50 were compared.

### Methane emission

The average daily amount of CH_4_ produced by the sheep decreased (*P* < 0.001) from 31 g without hazel leaves to 22 g per sheep fed with 50% hazel leaves in their diet. The decrease had linear (*P* < 0.001) and non-linear components (*P* < 0.05), where the reduction was particularly pronounced with 50% hazel leaves (Table 3). With the diet containing 50% hazel leaves together with PEG, the level of CH_4_ produced, though numerically higher, was not different from 50% hazel leaves without PEG, but different (*P* < 0.001) from the treatments with 25% hazel leaves and lucerne alone. The same patterns were found when relating CH_4_ to intakes of DM, OM and NDF. When scaling CH_4_ to BW^0.75^, to digestible DM, and to digestible OM, the effects of hazel leaves without PEG were similar, but the effect with the PEG-treated diet was no longer significantly different from the treatment with 25% hazel leaves. The CH_4_ release per unit of NDF digested did not differ between treatments, but a trend (*P* = 0.07) for a non-linear dosage effect was observed.

**Table 3:** Effect of hazel leaves on enteric methane emission of the sheep (n = 6).

Hazel leaves (% of diet)	Experimental diets				SEM	*P* values		
	0	25	50	50 + PEG		Diet	L^1^	Q^1^
Methane per day								
g/sheep	30.8^a^	29.4^a^	21.6^b^	24.9^b^	1.35	<0.001	<0.001	0.023
g/kg body weight^0.75^	1.20^a^	1.13^ab^	0.85^c^	0.97^bc^	0.045	<0.001	<0.001	0.054
Methane (g/kg intake of)								
Dry matter	15.6^a^	14.4^a^	10.5^b^	12.5^b^	0.50	<0.001	<0.001	0.003
Organic matter	17.9^a^	16.3^a^	11.6^b^	13.7^b^	0.60	<0.001	<0.001	0.003
Neutral detergent fibre (NDF)	32.0^a^	30.9^a^	21.6^b^	25.4^b^	1.02	<0.001	<0.001	0.002
Digestible dry matter	28.0^a^	26.7^a^	21.6^b^	25.3^ab^	0.85	0.007	0.004	0.085
Digestible organic matter	30.3^a^	28.5^a^	22.8^b^	26.5^ab^	0.92	0.002	<0.001	0.064
Digestible NDF	64.3	71.1	63.4	63.0	2.00	0.098	0.57	0.069

L, linear effect of hazel leaf proportion; PEG, polyethylene glycol; Q, quadratic effect of hazel leaf proportion; SEM, standard error of mean. Means carrying no common superscript are different at *P* < 0.05. ^1^For this analysis, only diets 0, 25 and 50 were compared.

### Nitrogen balance

The daily N intake linearly (*P* < 0.001) declined with an increasing proportion of hazel leaves (Table 4). At the same time, the absolute faecal N excretion linearly (*P* < 0.001) increased. This was accompanied by large and linear (*P* < 0.001) declines in the excretion of absolute urinary N and the proportion of urinary N of either total N ingested or total N excreted. The latter two variables also included a trend (*P* = 0.06) or a significant (*P* < 0.05) non-linear component where the 25% dosage of hazel leaves was proportionately less effective than the extra 25% hazel leaves in the diet with 50% hazel leaves. As a consequence of the increase in faecal N losses, the apparent N digestibility decreased along with the increased proportion of hazel leaves compared to lucerne alone in the pellets. This happened in a linear (*P* < 0.001) manner with a non-linear component (*P* < 0.05). Increasing the hazel leaf proportion from 0 to 50% in the diet decreased the body N retention (*P* < 0.001) and the utilisation of dietary N for the body N retention (*P* < 0.05). The addition of PEG to the diet with 50% hazel leaves further decreased (*P* < 0.001) the daily N intake, faecal N and the losses of faecal and urinary N, but increased (*P* < 0.001) urinary N compared to the non-PEG 50% diet. Adding PEG prevented (*P* < 0.001) the extra reduction in relative N losses from urine (per total N intake or total N excreted) and apparent N digestibility when increasing the hazel leaf proportion from 25% to 50%. The adverse effect on the relative loss of faecal N (but not faecal and urinary N, and the relative body N retention per total N intake) was alleviated (*P* < 0.05) by the addition of PEG.

**Table 4:** Effect of hazel leaves on nitrogen balance of the sheep (n = 6).

Hazel leaves (% of diet)	Experimental diets				SEM	*P* values		
	0	25	50	50 + PEG		Diet	L^1^	Q^1^
g/day								
Nitrogen (N) intake	62.4^a^	54.8^b^	49.3^c^	46.7^d^	1.69	<0.001	<0.001	0.44
Faecal N	24.9^bc^	28.7^b^	37.5^a^	23.9^c^	1.36	<0.001	<0.001	0.16
Urinary N	27.1^a^	18.1^b^	7.6^c^	16.0^b^	1.53	<0.001	<0.001	0.41
Faecal and urinary N	51.9^a^	46.8^b^	45.1^b^	39.9^c^	1.44	<0.001	0.005	0.33
Body N retention^2^	10.4^a^	8.1^ab^	4.3^c^	6.8^bc^	0.60	<0.001	<0.001	0.44
% of N intake								
Faecal N	39.9^c^	52.3^b^	75.7^a^	51.3^b^	2.80	<0.001	<0.001	0.022
Urinary N	43.4^a^	32.8^b^	15.3^c^	34.3^b^	2.18	<0.001	<0.001	0.062
Faecal and urinary N	83.3^b^	85.1^ab^	91.0^a^	85.6^ab^	0.96	0.015	0.008	0.35
Body N retention^2^	16.7^a^	14.9^ab^	9.0^b^	14.4^ab^	0.96	0.015	0.008	0.35
Apparent N digestibility (%)	60.1^a^	47.7^b^	24.3^c^	48.7^b^	2.80	<0.001	<0.001	0.022
Urinary N (% of N excreted)	52.1^a^	38.6^b^	16.8^c^	40.1^b^	2.71	<0.001	<0.001	0.028

L, linear effect of hazel leaf proportion; PEG, polyethylene glycol; Q, quadratic effect of hazel leaf proportion; SEM, standard error of mean. Means carrying no common superscript are different at *P* < 0.05. ^1^For this analysis, only diets 0, 25 and 50 were compared. ^2^Including N in wool grown during the periods.

### Energy balance

The supply with digestible energy and metabolisable energy (ME) decreased linearly (*P* < 0.001) with increasing proportions of hazel leaves (Table 5), although relative and absolute GE intake differed only marginally between treatments (Tables 2 and 5). This decrease was slightly more pronounced with the high compared with the low hazel leaf proportion (trends for non-linear effects) (Table 5). The faecal energy losses were increasing and those with urine and CH_4_ were declining with increasing hazel leaves proportion, all in a linear manner (*P* < 0.01) with a certain quadratic effect (*P* < 0.05) in the latter two. Heat energy losses were not affected by the treatments. For total energy losses, the overriding effects were faecal losses resulting in linearly (*P* < 0.01) increasing total losses. However, body energy retention also linearly declined (*P* < 0.05) with increasing hazel leaf proportions. As GE intakes did not differ much, the diet effects for relative energy turnover (per unit of GE intake) were similar to absolute energy losses. There was a decline by 11 percentage units in the apparent digestibility of GE when the hazel leaf proportion was increased from 0 to 50%. The decline had linear (*P* < 0.001) and by tendency non-linear components (*P* = 0.051). The decline in metabolisability was less pronounced in magnitude because part of the energy losses in faeces were counterbalanced by lower urinary and CH_4_ energy losses. The addition of PEG to the diet with 50% hazel leaves increased (*P < *0.001) the supply with digestible energy and ME. Adding PEG prevented (*P* < 0.001) part of the faecal energy loss per GE intake and the considerable decline in digestibility when increasing the hazel leaf proportion from 25% to 50%. No difference (*P* > 0.10) was observed between treatments in the absolute and relative heat energy losses of the sheep.

**Table 5:** Effect of hazel leaves on energy balance of sheep (n = 6).

Hazel leaves (% of diet)	Experimental diets				SEM	*P* values		
	0	25	50	50 + PEG		Diet	L^1^	Q^1^
Energy intake (MJ/day)								
Gross energy (GE)	37.1^b^	37.4^ab^	38.6^ab^	39.2^a^	0.81	0.03	0.009	0.68
Digestible energy	20.6^a^	19.6^ab^	17.2^c^	18.8^b^	0.47	<0.001	<0.001	0.058
Metabolisable energy^2^ (ME)	17.4^a^	16.8^ab^	14.8^c^	16.0^b^	0.38	<0.001	<0.001	0.078
Energy loss (MJ/day)								
Faeces	16.4^b^	17.7^b^	21.4^a^	20.4^a^	0.63	<0.001	<0.001	0.14
Urine^3^	1.56^a^	1.21^b^	1.22^b^	1.48^ab^	0.056	0.007	0.005	0.047
Methane^4^	1.70^a^	1.63^a^	1.19^b^	1.37^b^	0.075	<0.001	<0.001	0.023
Heat^5^	10.6	10.4	9.9	10.8	0.33	0.72	0.56	0.69
Total energy loss	30.3^b^	30.9^b^	33.7^a^	34.1^a^	0.84	0.001	0.003	0.33
Body energy retention^6^	6.73^(a)^	6.42^(ab)^	4.90^(b)^	5.19^(ab)^	0.334	0.055	0.041	0.44
Energy turnover (% of GE intake)								
Faeces	44.2^d^	47.5^c^	55.3^a^	52.0^b^	0.96	<0.001	<0.001	0.051
Urine	4.19^a^	3.25^b^	3.17^b^	3.78^ab^	0.128	0.005	0.002	0.039
Methane	4.59^a^	4.35^a^	3.10^b^	3.48^b^	0.178	<0.001	<0.001	0.039
Heat	28.7	27.7	25.6	27.5	0.68	0.67	0.26	0.71
Total energy loss	81.7^b^	82.8^ab^	87.2^a^	86.8^ab^	0.92	0.032	0.025	0.47
Body energy retention	18.3^a^	17.2^ab^	12.8^b^	13.2^ab^	0.92	0.032	0.025	0.47
Heat energy (% of ME intake)	61.6	61.7	66.8	67.6	1.72	0.31	0.13	0.60
Apparent digestibility (% of GE)	55.8^a^	52.5^b^	44.7^d^	48.0^c^	0.96	<0.001	<0.001	0.051
Metabolisability^7^ (% of GE)	47.0^a^	44.9^a^	38.4^b^	40.7^b^	0.80	<0.001	<0.001	0.084

L, linear effect of hazel leaf proportion; PEG, polyethylene glycol; Q, quadratic effect of hazel leaf proportion; SEM, standard error of mean. Means carrying no common superscript without or with brackets are different at *P* < 0.05 and *P* < 0.10, respectively. ^1^For this analysis, only diets 0, 25 and 50 were compared. ^2^Metabolisable energy (ME; MJ/day) = GE intake (MJ/day) − faecal energy loss (MJ/day) − CH_4_ energy loss (MJ/day) − urinary energy loss (MJ/day). ^3^Urinary energy^46^ (MJ/day) = 0.0348 × urinary carbon (g/day) + 0.009 × urinary N (g/day). ^4^CH_4_ energy^47^ (MJ/day) = CH_4_ (l/day) × 0.03957. ^5^Heat energy^48^ (MJ/day; corrected for assumed CO_2_ production from microbial fermentation) = 0.01618 × O_2_ (l/day) + 0.00502 × [CO_2_ (l/day) − 3 × CH_4_ (l/day)] − 0.00217 × CH_4_ (l/day) − 0.00599 × urinary N (g/day). ^6^Body energy retention (MJ/day) = ME (MJ/day) − heat energy loss (MJ/day). ^7^Metabolisability (% of GE) = ME intake (MJ/day)/GE intake (MJ/day) × 100.

## Discussion

### Forage value of hazel leaves

Little information is available regarding the forage value of hazel leaves as feed for ruminant livestock. To our knowledge, the current experiment is the first to provide data on the feed intake, nutrient digestibility, as well as the N and energy balance of sheep fed hazel leaves in comparison with a known high quality forage. The amounts of 25 and 50% hazel leaves included in the diets were substantial and were intended to provoke the expression of adverse effects on nutrient intake and digestibility if there were any.

Compared to the lucerne (treatment 0), the hazel leaves were characterised by 39% lower CP contents (being excessive for ruminants in the lucerne), quite similar in fibre contents, and 28% higher lignin contents. The addition of PEG to the pellets should not have caused a decline in the content of phenolic compounds due to maintaining the hazel leaf proportion. However, according to the analysis, the pellets with PEG appeared to contain fewer TP, total tannins and CT than the corresponding pellets without PEG. As the difference in CT content was particularly large, stable bonds between CT and PEG likely compromised the CT extraction applied in the laboratory analysis. Therefore, for the PEG-containing pellets, the ratios of PEG: CT and PEG: total tannins were calculated based on the CT and total tannin contents measured in the PEG-free pellets.

One important constraint in employing tanniferous feeds in ruminant nutrition is their often low palatability, which may either limit the intake of these feeds below the levels intended or, especially when mixed with the rest of the diet, impair the overall feed intake. This was not the case in the present study, even when feeding the hazel leaves in a dietary proportion of 50%. It was reported that moderate amounts of tannins (depending on the type of tannins) may improve digestion and animal performance without affecting the voluntary feed intake^11^. When exceeding a CT concentration of 5 to 6% in dietary DM, a reduction in feed palatability and thus feed intake is commonly observed^24,25^. In the present experiment, the dietary CT contents of about 2% in DM remained far below this threshold. However, the restricted access to the diet and mixing the hazel leaves with lucerne might have also prevented differences in DM intake, which could have occurred under conditions of either *ad libitum* access or separate presentation of the hazel leaves. A second constraint in feeding tanniferous feeds often involves adverse effects on nutrient digestibility. Only in cases of dietary CP, a decreased ruminal digestion is favourable, provided the tannin-CP bonds are later cleaved and the protein can be digested in the lower gut. In the present study, the apparent total tract digestibility of OM, and especially of NDF, ADF and N, was reduced when the sheep consumed pellets with increasing proportions of hazel leaves. As lucerne is a high-quality forage, the effect on digestion might in part be the consequence of the comparably lower digestibility of the hazel leaves, an assumption supported by the higher lignin content of the hazel leaves compared to lucerne (12.2 vs. 9.5% of DM). Lignin is the diet constituent most adverse to fibre digestion by the ruminant^26^. However, the difference in lignin content was not sufficiently high to serve as the only explanation. The tannins present in the hazel leaves may have additionally impaired digestibility. Tannins not only bind to protein but also to carbohydrates although to a lesser extent^11,27^, thus slowing down their ruminal degradation. Tannins are also known to lower the activity of fibrolytic enzymes, an effect depending on the dose and type of tannins^11,28,29^. Accordingly, approximately 4% of tannins in the diet are sufficient to lower digestibility of OM, NDF and ADF, as Carulla *et al*.^17^ found in growing lambs when using *Acacia mearnsii* extract and Al-Kindi *et al*.^28^ identified in goats when using quebracho tannin extract. Indeed, the addition of PEG to the diet containing 50% hazel leaves alleviated the adverse effect of the hazel leaves on digestibility, but this only partially and not fully. A PEG: CT ratio of 1.6:1 in the diet should have been sufficiently high to prevent the bioactivity of the CT in the rumen^30^. However, still not all dietary tannins might have been inactivated by the PEG because, when also considering the hydrolysable tannins which made up half of the total tannins, the PEG: total tannin ratio was only 0.8:1. It has been described in the literature^22^ that PEG can also form complexes with HT. In addition, it cannot be excluded that other active ingredients impaired digestibility, such as the non-tannin phenols that made up to 28% of dietary TP. Finally, the low fibre digestibility with the high proportion of hazel leaves may have been indirectly caused by the inactivation of dietary protein in the rumen by the CT. This might have resulted in a deficiency of the supply of rumen-degradable protein (RDP). A deficiency of RDP is especially impairing to the activity of the fibre degrading rumen microbes^31^. However, as the decline with increasing hazel leaf proportions was mostly linear for OM and NDF digestion and, therefore, also occurred with the lower level of leaves, a RDP deficiency with the high leaf proportion seems unlikely. Nevertheless, further studies are needed to determine whether the hazel leaf effects are independent from the level of supply with RDP. It also must be stated that according to Makkar *et al*.^32^ the analysis of the fibre digestibility of tanniferous feeds using the detergent extraction techniques, as applied in the present study, is not always accurate in the presence of tannins and PEG.

Consistent with the lower digestibility, the addition of the hazel leaves also resulted in lower energy and protein retention in the body of the sheep and, again, part of this effect was compensated for by adding PEG. The results agree well with those of a previous study by Tiemann *et al*.^24^ in which digestible energy and metabolisable energy were adversely affected by increasing the proportion of the CT-rich foliage of *Calliandra calothyrsus* and *Flemingia macrophylla* in the diet of sheep, where also fibre digestibility was reduced. Nevertheless, the efficiency of energy utilization in the metabolism was obviously not impaired, as the treatments had no significant effect on heat energy loss in relation to GE intake. That means that the substrates absorbed from the gut with the four experimental diets appear to have been similarly valuable to the animal. The use of a tanninferous forage like the hazel leaves could be advantageous for metabolic protein utilisation by the animals because of the proposed increase in ruminal bypass feed protein^14^ and in the absorption of essential amino acids from the intestine in the presence of tannins (reviewed by Min *et al*.^33^). Still, body N (protein) retention declined with increasing hazel leaf proportions from about 10 to 4 g/day, consistent with the negative effect on the apparent total tract N digestibility found in the present study and elsewhere^17,28^. This indicates that the cleavage of the tannin-protein bonds in the lower gut was not efficient enough to maintain or improve the metabolic supply with protein and amino acids. Whether or not the tannin-protein complexes protected from ruminal degradation are cleaved depends on the type of tannin, its chemical structure and structural flexibility^34^ as well as on the pH of the gut environment^11^. The presence of tannins may even enhance endogenous N losses, leaving the extent to which the increased faecal N losses are really caused by a decreased true N digestibility unclear^17^.

### Effects of hazel leaves on methane emission

The addition of hazel leaves to the diet clearly depressed CH_4_ emissions. This included both the absolute CH_4_ emission per head per day and the relative CH_4_ emission per intake as well as per BW^0.75^. The present study, with levels of CH_4_ mitigation ranging from 25% (per unit of digestible OM) to 35% (per unit of OM intake) in live animals, confirms previous *in vitro* results^20,21^. The level of CH_4_ mitigation per unit of digestible OM of 6% found in the present study at a dietary tannin content of 2.4% was exactly as predicted by the equation of Jayanegara *et al*.^35^ for *in vivo* studies, but for 4% dietary tannins the mitigation level found was far higher with 25% than the predicted 11%. Similarly, Archimède *et al*.^36^ observed a decline by 29% in the CH_4_ emission per unit of digestible OM of sheep when adding cassava (*Manihot esculenta*) leaves to a grass-based diet at a level equivalent to 4% tannins in dietary DM. Carulla *et al*.^17^ found that 4% tannins from *Acacia mearnsii* could decrease methanogenesis per unit of digestible OM by 7% from sheep. As with digestibility, the addition of PEG counterbalanced part of the effect, indicating that hazel leaf tannins directly or indirectly lowered methanogenesis. The mode of action by which tannins mitigate CH_4_ emission from ruminants is not entirely understood. The most accepted explanations are the concept of substrate deprivation, such as the reduction in fibre digestion, which decreases H_2_ production being available for reducing carbon dioxide (CO_2_) to CH_4_, and the direct inhibition of the growth of the methanogens^7^. In the present study, the CH_4_ produced per unit of NDF digested was not affected by the supplementation with hazel leaves, at least not in a linear manner. This lack of response is in agreement with observations made by Carulla *et al*.^17^. However, a direct reducing effect of hazel leaves on either methanogens or other ruminal microorganisms, such as the protozoa, cannot be fully excluded. In addition, when employing intact plant parts as diet components, other ingredients apart from the tannins, such as lipids, saponins and essential oils, may be bioactive^35^. This was not examined in the present study.

### Effects of hazel leaves on urinary nitrogen excretion

The traits affected most intensively by the dietary treatments were those related to urinary N excretion. Urinary N is the main way of removal of excessive metabolic N from the body of ruminants. However, it is also the major source of ammonia emission from stored manure, leading to air and water pollution, soil acidity and fine particulate matter formation^37,38^ and of nitrous oxide emission from manure-amended soil^38^. In the present study, replacing lucerne with hazel leaves was effective to reduce the metabolic N load in two ways. First, due to the lower N content of the hazel leaves, the excessive N intake from the lucerne was substantially reduced. Lucerne protein is highly ruminally degradable and promotes absorption through the rumen wall as ammonia and is disposed via urine^11^. Accordingly, along with a decreasing proportion of lucerne in the pellet (from 100 to 38% in the PEG-supplemented pellet), and thus decreasing N intake, the urinary N excretion per unit of N intake decreased, whereas the faecal N excretion per unit of N intake increased. This modified the excretory pattern (urinary N:faecal N) from about 1:1 to 0.7:1. Second, the tannins present in the leaves depressed the net N absorption from the gut. It was not possible to separate these two factors of influence in the present study, even when relating urinary N excretion to N intake. Still, the present results reflect the current feeding practice where a high quality, highly fertilised forage grass or legume is complemented by tanniferous plant material like the hazel leaves. In combination, the absolute urinary N excretion was reduced by up to 72%, and the reduction relative to N intake was still as great as 28 percentage units. This caused a significant shift of N excretion from urine to faeces, which was clearly less when PEG was added in the present study. When stored as slurry, gaseous N emissions would have been drastically reduced by this feeding measure^39^. Therefore, the substantial shift in N excretion from urine to faeces when feeding hazel leaves is highly favourable from an environmental perspective, as gaseous N emission and N leaching potentials are considerably reduced. The lower amount of N to be disposed via urine also led to a decrease in water consumption (−28%) and urine volume (1.2 vs. 2.3 kg per day; data not shown in the table) for the diet with 50% compared to that with 0% hazel leaves, respectively. Tannin supplementation was previously found to be effective in shifting urinary N to faecal N, as in the study of Carulla *et al*.^17^ where 4% *Acacia mearnsii* tannin extract in DM decreased urinary N and increased the faecal N excretion per N intake by 14% and 30%, respectively.

An application of hazel leaves in ruminant nutrition on a broad scale would require ample amounts of hazel leaves. This would require the joint impetus of farmers, consumers and politicians to produce and consume climate-friendly foods from livestock. In addition, an extended cultivation of this woody plant would increase biodiversity, also by providing a habitat for numerous species.

### Conclusion

Hazel leaves, found promising in mitigating CH_4_ emission from *in vitro* rumen fermentation screenings, indeed turned out to be very efficient in live animals receiving high hazel levels. To be implemented in feeding practice, the forage value in terms of intake (or palatability) and digestibility may not be compromised. In the present study, dietary intake was sustained, even with high levels of hazel leaves replacing the high-quality forage lucerne. However, organic matter digestibility and body energy and N retention were lower with diets containing high levels of hazel leaves. This limitation is theoretically easy to overcome, for instance by adding diet ingredients rich in rumen fermentable energy, providing both metabolisable energy and protein (from microbes). However, this needs to be ascertained in further experiments as rumen fermentation and CH_4_ emission may be affected by this dietary modification as well. The unchanged intake indicating a high palatability of hazel leaf supplements provides an advantage over many other plant-based supplements.

## Methods

### Experimental diets

Diets were composed of hay (late cut, ryegrass-dominated), dehydrated hazel leaves and lucerne. Hazel leaves were purchased from Alfred Galke GmbH (Bad Grund, Germany) and lucerne (*Medicago sativa*) from Landi (Sense-Düdingen, Switzerland). Hazel leaves and lucerne were harvested in Albania in 2015 and in France (Marne) in 2016, respectively. Four types of experimental pellets were produced from lucerne and hazel leaves containing 0%, 30% and 60% hazel leaves, the latter also with the addition of 3.8% PEG (molecular weight of 6000; Sigma, St. Louis, MO, USA) on a DM basis (Table 1). The PEG replaced lucerne in the pellets to maintain the dietary content of phenolic compounds from hazel. The realised dietary PEG: CT and PEG: TT ratios were 1.6:1 and 0.8:1, respectively. Hazel leaves as purchased had a cutting size of 4 to 6 mm. Lucerne was chopped to a size of 3 mm using a hammer mill (Sigma 5.2, Kuhn AG, Bottighofen, Switzerland). Chopped hazel leaves and lucerne were mixed with a batch mixer (Speedmix DFML-1000, Bühler AG, Uzwil, Switzerland) and subsequently pelleted (Kahl 40PS, Amandus Kahl GmbH & Co, Reinbek, Germany) with the use of steam (about 60 °C; Installation Bühler AG, Uzwil, Switzerland). Pellets had a diameter of 4.5 mm. The complete diets consisted of hay and experimental pellets at a ratio of 20%: 80% in DM, resulting in hazel leaf proportions of approximately 0, 25 and 50% in the total diet (realised: 0, 23.4 and 46.8%). The amounts of diets offered provided 1.6 times the recommended DM supply for the maintenance requirements of adult female sheep^40^. The pellets were not balanced for CP because the CP content in the complete diets (≥13.2% CP in DM) was always higher than the threshold where the RDP supply is assumed to become critical^31^, although this did not consider possible RDP declines by tannin-protein bonds. Half of the daily portion of the experimental pellets was fed at 08:00 h and the other half at 15:00 h. Around 30 min later, the corresponding proportion of the hay was offered, a time when most to all of the pellets had been consumed. The animals had free access to water.

### Animals and experimental design

The experimental protocol complied with the Swiss legislation for Animal Welfare and was approved (ZH 25/16) by the Committee on Animal Experimentation of the Cantonal Veterinary Office Zurich. Six female non-lactating Swiss Black-Brown Mountain sheep with an initial age of 18 ± 1.7 months and an average BW of 71 ± 5.7 kg were used from August 2016 to January 2017. No worm treatment was performed, as the faecal egg count showed that the animals were free of worms. Animals were fed lucerne-only pellets and hay during 8 days before the main experiment started. The six sheep were allocated to different sequences of four experimental diets in four subsequent 18-day periods in a 6 × 4 crossover design. The animals were weighed after the morning feeding on Days 1, 12 and 19, using data from Day 12 as the reference value for defining daily DM supply on Days 12 to 18. To resume social grouping behaviour and to minimize the carry-over effects of the previous treatment to the following treatment, periods were separated by 2 days of washout period when the animals were group-housed and fed the lucerne-only pellets and hay but no lucerne + hazel leaves pellets. Each period was divided into 11 days (Days 1 to 11) of adaptation to diets in individual pens (size of 1.25 × 2.5 m, floor covered with sawdust) and 7 days (Days 12 to 18) of complete collection of faeces and urine, including 2 days of individual gaseous exchange measurements performed subsequently with two sheep in parallel. For the 7 days of sample collection and gaseous exchange measurements, the sheep were transferred into metabolic crates (floor area 2 m^2^) with transparent acrylic glass side panels allowing visual contact. The animals were tethered at the front and the floor was covered with a rubber mattress. The front part was non-perforated, and the rear part was grated (column width 20 mm/beam width 40 mm) allowing faeces and urine to fall into the funnel beneath. A screen (beam width 4 mm) in the funnel retained faeces, but not urine. The daily intake of hay and experimental pellets was recorded by weighing amounts offered and refused. Between Days 12 and 18, individual hay leftovers were sampled and water consumption and the amounts of faeces and urine excreted were recorded. A proportion of 10% of total faeces was collected daily and frozen at −20 °C. The urine was ducted into two containers, one for untreated urine and the other containing 7.1 mol/l sulphuric acid to inhibit N evaporation. Urine from each container was collected daily in proportions of 0.5% of the amounts excreted and stored at −20 °C. Later all types of excreta samples were composited per animal per period. Part of the faeces was dried at 60 °C to a constant weight. The hay was sampled four times and the pellets two times. Hay leftovers were pooled across animals per period.

For the gaseous exchange measurements, two sheep at a time were transferred to two open-circuit respiration chambers (8.3 m^3^ each)^41^. For the feeding, the chambers were entered via an airlock. From the total stay of 48 h in the chambers, data of 1 to 1.7 h had to be interpolated from adjacent values, as the measurement was then interrupted for cleaning purpose. The chambers were air-conditioned, and the temperature, relative humidity, air pressure and airflow were set to 18 °C, 70%, −60 Pa and 400 l/min (Promethion FG-1000 flow generators, Sable Systems Europe GmbH, Berlin, Germany), respectively. Lighting conditions were diurnal (7 h lights on, 19 h lights off). The concentrations of CH_4_, CO_2_ and O_2_ were measured in each respiration chamber every 3 min for 1 min with a gas analyser (Promethion GA-4, Sable Systems). Prior to gaseous exchange measurement, the gas analyser was calibrated automatically using pure N_2_ (99.999%) and a mixed gas (19.8% O_2_, 1.0% CO_2_, 0.1% CH_4_, in N_2_ as carrier). Recovery was tested before each experimental period by burning propane gas. The mean recovery was 95%.

### Laboratory analyses

Feed pellets and dried faeces were ground with a centrifugal mill (1-mm screen) and hay with a cutting mill (1-mm screen). Feeds, refusals and dried faeces were analysed for DM, total ash (TGA 701, Leco Corporation, St Joseph, MI, USA; AOAC^42^ index no. 942.05), NDF, ADF and acid detergent lignin (Fibertec System M 1020 Hot Extractor and 1021 Cold Extractor; Tecator, Höganäs, Sweden). Fibre data were corrected for ash content, and heat stable α-amylase was used for the NDF analysis, but no sodium sulphite was added^43^. Acid detergent lignin was determined sequentially after the ADF analysis by digestion in sulphuric acid (72%) for 3 h. The content of GE was determined using a Calorimeter (C7000, IKA-Werke GmbH & Co. KG, Staufen, Germany). The N content of feeds, refusals, non-dried faeces and acidified urine was analysed with a C/N analyser (TruMac CN, Leco Corporation, St. Joseph, Michigan, USA; AOAC index no. 968.06). Crude protein (CP) was calculated as 6.25 × N. The carbon content of the non-acidified urine was determined with the same analyser. The feeds were analysed for ether extract (extraction System B-811, Büchi, Flawil, Switzerland; AOAC index no. 963.15). Total phenols in feed items were extracted by 70% acetone based on Makkar^44^ with modifications described in Jayanegara *et al*.^45^. In brief, a modified Folin-Ciocalteu method was applied for TP and non-tannin phenols and data were expressed as gallic acid equivalents (Sigma, St. Louis, MO, USA). The CT were measured using the butanol-hydrochloride-iron method, and the values were expressed as leucocyanidin equivalents^44,45^. The contents of total tannins and HT were calculated as the difference between TP and non-tannin phenols, and between total tannins and CT, respectively.

### Calculations and Statistical Analyses

The equations used to calculate the energy-balance related variables are specified in the footnote to Table 5. Individual feed intake, digestibility, N and energy balance were calculated based on the data combined across the 7 day collection periods and, in the case of gas exchange data, across the 2 days of respiration chamber measurements. The BW is given as the average of Days 1 and 19 of each period. To relate variables to BW^0.75^, BW means of measurements made on Days 12 and 19 of each period were used. Data were subjected to analysis of variance with the Mixed procedure of SAS (version 9.4, SAS Institute, Cary, NC), considering the treatment and the period as fixed effects and animals as the random effect. Pairwise multiple comparisons among the least-square means were performed by the Tukey-Kramer test. Linear and quadratic effects of the level of hazel leaves (0%, 25% and 50%) were evaluated by orthogonal polynomial contrasts. Differences were considered statistically significant at *P* < 0.05 and trends at 0.05 ≤ *P* < 0.10.

### Data availability

All data generated or analysed in the present study are included in this published article.
